# Intermittent Exposure to Aflatoxin B_1_ Did Not Affect Neurobehavioral Parameters and Biochemical Markers of Oxidative Stress

**DOI:** 10.3390/brainsci13030386

**Published:** 2023-02-23

**Authors:** Ana Claudia Monteiro Braga, Naieli Schiefelbein Souto, Fernanda Licker Cabral, Micheli Dassi, Érica Vanessa Furlan Rosa, Naiara dos Santos Guarda, Luiz Fernando Freire Royes, Michele Rechia Fighera, Rafael Noal Moresco, Mauro Schneider Oliveira, Marcel Henrique Marcondes Sari, Ana Flávia Furian

**Affiliations:** 1 Programa de Pós-Graduação em Farmacologia, Universidade Federal de Santa Maria, Santa Maria 97105-900, Brazil; 2 Programa de Pós-Graduação em Ciência e Tecnologia dos Alimentos, Universidade Federal de Santa Maria, Santa Maria 97105-900, Brazil; 3 Programa de Pós-Graduação em Ciências Farmacêuticas, Universidade Federal de Santa Maria, Santa Maria 97105-900, Brazil; 4 Programa de Pós-Graduação em Ciências Biológicas: Bioquímica Toxicológica, Universidade Federal de Santa Maria, Santa Maria 97105-900, Brazil

**Keywords:** aflatoxin B_1_, intermittent exposure, oxidative stress, behavior, irregular consumption

## Abstract

Aflatoxin B_1_ (AFB_1_) is the most common toxic mycotoxin that contaminates food. The treatment of its intoxication and the management of contaminations are a constant subject of health agendas worldwide. However, such efforts are not always enough to avoid population intoxication. Our objective was to investigate whether intermittent exposure to AFB_1_ would cause any impairment in biochemical and behavioral parameters, intending to simulate an irregular consumption. Male Wistar rats received four AFB_1_ administrations (250 μg/kg) by intragastric route separated by a 96-h interval. Toxicity was evaluated using behavioral tests (open field, object recognition, nest construction, marble burying, and splash test), biochemical markers of oxidative stress (cerebral cortex, hippocampus, liver, and kidneys), and plasma parameters of hepatic and renal functions. The intermittent exposure caused no modification in body weight gain as well as in organ weight. Both control and AFB_1_ groups presented similar profiles of behavior to all tests performed. Furthermore, AFB_1_ administrations alter neither antioxidant defenses nor markers of oxidation in all assayed tissues and in the plasma markers of hepatic and renal functions. Therefore, AFB_1_ intermittent administration did not cause its common damage from exposure to this toxicant, which must be avoided, and additional studies are required.

## 1. Introduction

Mycotoxins are fungi secondary metabolites and one of the main food safety problems, due to their effects on human and animal health [1]. AFB_1_ is the most common toxic mycotoxin known, found mainly in oilseeds, namely soybean, sunflower, almond, chestnut, peanut, as well as in spices, dried fruits, and beans [2]. In addition, all products derived from these raw materials are subject to perpetuating contamination, since AFB_1_ is very stable and resistant, difficult to eliminate, and toxic even at low concentrations [3,4].

AFB_1_ is absorbed in the gastrointestinal tract and metabolized in the liver by cytochrome P450 enzymes in a toxic metabolite (AFB_1_-8,9-epoxide). Additionally, AFB_1_ is classified as a human carcinogen I (International Agency for Research on Cancer) [5] and is known to induce a variety of biological acute toxicity, teratogenicity, mutagenicity, impaired growth, immunosuppression, genotoxicity, increased lipid peroxidation, and free radical generation, as well as changes in the central nervous system [6], posing a threat to public health, especially in developing countries [7].

The consumption of food commonly contaminated by AFB_1_ is increasing mainly considering the so-called “healthy” trend—transition from stage 4 to stage 5 of food evolution—that stimulates the consumption of whole grains, where this mycotoxin is frequently found [8]. This change in feeding behavior directly influences exposure to AFB_1_, especially since the maximum threshold allowed by developing country legislation is up to seven times that of developed countries, and one-quarter of the cereals produced are contaminated with mycotoxins [9].

To date, the main ways of controlling AFB_1_ intoxication are prevention before consumption and detoxification after consumption [1]. Methods of detection and biodegradation are known and developed; however, they involve an expensive technology, hindering the access and their application [10,11,12].

Literature shows exposure protocols for elucidation, reversion, or protection from AFB_1_-induced damages [13,14,15,16]. Nevertheless, studies investigating exposure strategies that avoid or decrease the damages identified in the population are scarce. Most of the protocols tested repeated exposure [13,17,18]. Nonetheless, if it was already demonstrated that single, acute, sub-chronic, and chronic exposure leads to aggravations and do not have the option of stopping the consumption of contaminated foods, alternatives should be sought.

Based on previous experiments performed by our research group and others [14,15,18,19], instead of a single or repeated exposure, our proposal was to perform different times of exposures, separated by a 96-h non-tested interval. The approach aims to investigate whether intermittent exposure over a period would be sufficient to guarantee the homeostasis of the organism. Thus, we sought to evaluate whether AFB_1_ toxicity in rats would be mitigated using a protocol of intermittent exposure. For this aim, general toxicity signals were determined by behavioral, biochemical, and molecular approaches.

## 2. Materials and Methods

### 2.1. Chemicals and Animals

AFB_1_ (Cas No. 1162-65-8, ≥95% purity, Cayman Chemical, Ann Arbor, MI, USA) was dissolved in 2% DMSO at a final concentration of 250 µg/mL. All other chemicals used were of pure analytical grade and were from standard commercial suppliers.

This study was conducted using sixteen young male Wistar rats (53.31 ± 3.281 g). The animals were maintained in cages of polypropylene (41 cm × 34 cm × 16 cm L × W × H, 1394 cm^2^) under a 12:12 h light-dark cycle, with lights turned on at 07:00 a.m.; environmental controlled temperature (24 ± 1 °C) and relative humidity (45–65%); and had free access to water and food. At the end of the protocol, prior to tissue collection, the animals were euthanized by decapitation in a hand-operated guillotine, which was performed by an expert researcher. The experimental protocol was approved by the Ethics Committee for Animal Research of the Federal University of Santa Maria, Brazil (Approval number 5166290316/2016) and carried out in strict accordance with the recommendations in the national and international legislation (Brazilian Council of Animal Experimentation guidelines—CONCEA—and of U.S. Public Health Service’s Policy on Humane Care and Use of Laboratory Animals—PHS Policy).

### 2.2. Experimental Design

**Figure 1:**
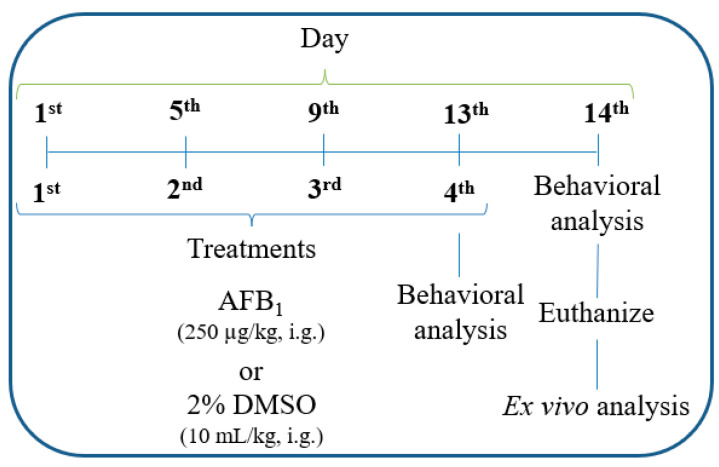
Schematic representation of the experimental design. Animals were divided into two groups (AFB_1_ and DMSO) and treated with four doses separated by an interval of 96 h between each dose.

The experimental design is illustrated in Figure 1. The animals were randomly divided into two groups. The control group received four doses of 2% DMSO (10 mL/kg, b.w.), while the AFB_1_ group received four administrations of AFB_1_ (250 µg/kg, b.w.). The doses were administered by the intragastric route and separated by an interval of 96 h among the treatments. It was based on previous studies that showed AFB_1_ intoxication signals after smaller intervals of exposure [14,15,18,19]. Animals’ body weight was monitored over the experimental period. The behavioral tests were conducted after the last dose and the next day was followed by euthanasia for tissue collection. The blood samples were obtained after decapitating the animals, by collecting the tissue from the body trunk in heparinized vials for analysis. The samples were centrifugated for yielding plasma fraction, which was used for biochemical assays. The brain, heart, kidneys, liver, lungs, spleen, and testicles were weighed using an analytical scale to evaluate weight toxicity parameters. As concerns sample storage, brain samples (which were dissected for collecting the cerebral cortex and hippocampus) and kidney and liver samples were stored (−80 °C) for ex vivo evaluations.

### 2.3. Behavioral Analysis

On the 13th day, animals performed object recognition, open-field test, marble burying, and nest construction tests. On the 14th day, nest construction was evaluated, and object recognition was performed (long-term memory—24 h after training section), followed by the splash test (Figure 1). Behavioral analyses were conducted during the light cycle, between 9 a.m. and 3 p.m., except during the nest construction test that was carried out over the dark cycle (7 p.m. to 7 a.m.).

#### 2.3.1. Object Recognition Test

The object recognition test (ORT) was performed by submitting the animals to three experimental sessions, respectively, training #1 (first session—two equal objects [A1 and A2]), short-term memory assessment #2 (second session, 4 h after training #1—two distinct objects [A1 and B]), and long-term memory evaluation (third session, 24 h after training #1—two distinct objects [A1 and C]) in the same apparatus used to perform the open field test (Section 2.3.2). The animals were individually submitted to three sessions and for 10 min the time spent exploring each object was recorded. The results were expressed as a recognition index, which was calculated as previously described [20].

#### 2.3.2. Open Field Test

Animals were tested for 10 min in a round open field, where the floor was divided into ten equal parts. Five parts, which were near the walls, were considered the peripheral area and the rest was considered the central area. Immobility, crossings, rearing, and time spent in the central area were recorded and analyzed to evaluate the locomotor activity and anxiety-like behavior [20]. The results were expressed as the total number of crossings and rearing. The time spent in central areas was reported in seconds.

#### 2.3.3. Marble Burying Test

The marble burying test was conducted in individual cages filled with 5 cm of wood chip bedding and twelve marbles equidistant in a 2 × 6 arrangement. Then, after 30 min, the number of buried marbles (>50% marble covered by bedding material) was recorded [21].

#### 2.3.4. Nest Construction Test

Rats were allocated in individual cages covered with normal bedding and with a pressed cotton square (±3 g). Twelve hours later (dark cycle), the nest construction was evaluated and a score was attributed, as previously reported by Deacon and collaborators [22].

#### 2.3.5. Splash Test

Animals were individually observed over 10 min after a squirting of a 10% sucrose solution on the dorsal coat. The time to start the grooming behavior and the time of grooming were evaluated as an index of self-care and motivational behavior [23]. The results were expressed in seconds.

### 2.4. Ex Vivo Analysis

The blood collected was centrifuged at room temperature for 10 min to obtain the plasma fraction, which was used for assessing aspartate and alanine aminotransferase activity, creatinine, and albumin content, thiobarbituric acid reactive substances (TBARS) levels, and ferric reducing antioxidant power (FRAP).

Liver, kidney, cerebral cortex, and hippocampus samples were homogenized (1:10, *w*/*v*; 30 mM Tris-HCl pH 7.4) and centrifuged at 2400× *g* for 15 min at 4 °C to yield the supernatant fraction (S_1_), which was used for the biochemical analysis immediately after euthanization—protein, non-protein thiols (NPSH), ascorbic acid (AA), TBARS, and FRAP determination and Na^+^, K^+^-ATPase, catalase (CAT) and glutathione S-transferase (GST) activity. The other hemisphere of the cerebral cortex and hippocampus were stored at −80 °C for a Western blot analysis—protein kinase C (PKC) immunoreactivity.

#### 2.4.1. Plasma Biochemical Analysis 

Plasma levels of alanine aminotransferase (ALT), aspartate aminotransferase (AST), creatinine (CREA), and albumin (ALB) were evaluated in BS 380 Mindray Chemistry Analyzer (Shenzhen, China) using commercial kits (Bioclin, Brazil).

#### 2.4.2. Protein Determination

Protein content was determined by the Bradford method [24], using bovine serum albumin (1 mg/mL) as the analytical standard.

#### 2.4.3. NPSH Levels

The NPSH levels were determined according to the method proposed by Ellman [25]. Samples of S_1_ were precipitated using 10% trichloroacetic acid (TCA) and subsequently centrifuged at 10,000 rpm for 10 min to yield the supernatant fraction (S_2_). An aliquot of S_2_ (100 μL) was mixed with potassium phosphate buffer (1 M, pH 7.4) and 5,5′-dithio-bis-(2-nitrobenzoic acid (DTNB) (10 mM). The NPSH levels were measured at 412 nm using a spectrophotometer, and the results were expressed as nmol NPSH/mg of protein.

#### 2.4.4. Ascorbic Acid (AA) Levels

Samples were precipitated with 5% TCA and centrifuged at 3000 rpm for 10 min for ascorbic acid determination. Then, 100 µL of the supernatant was incubated with 13.3% TCA and a color reagent containing dinitrophenyl hydrazine, thiourea, and CuSO_4_, at 37 °C for 3 h. The reaction was stopped with 65% H_2_SO_4_ (*v*/*v*) and the system was measured at 520 nm in a spectrophotometer. The results were expressed as nmol ascorbic acid/mg of protein [26].

#### 2.4.5. TBARS Levels

During the lipid peroxidation process, one of the products formed is the malondialdehyde (MDA). This product reacts with thiobarbituric acid to form a colored complex, which is quantified by its absorbance. The color formed was measured at 532 nm using a spectrophotometer and the results were expressed as nmol MDA/mg of protein [27].

#### 2.4.6. FRAP Potential

This test is based on the reduction of the ferric 2,4,6-tripyridyl-s-triazine complex ([Fe (III)—(TPTZ)_2_]^3+^ in the intense blue iron complex [Fe (II)—(TPTZ)_2_]^2+^ by the action of antioxidant compounds present in the sample, including mainly uric acid, and vitamins C and E. The color formed is measured spectrophotometrically at 593 nm and the results are expressed as nmol Fe^2+^/mg of protein [28].

#### 2.4.7. Na^+^, K^+^-ATPase Activity

In this protocol, we evaluated in the cerebral cortex whether AFB_1_ caused changes in individual Na^+^, K^+^-ATPase α isoforms. For this, we used a classical pharmacological approach based on the isoform-specific sensitivity to ouabain. With a concentration of 12 µM ouabain, the isoform α1 is inhibited and then we discovered the activity of α2/α3 isoforms. Whereas, with a concentration of 8 mM ouabain, we inhibited the activity of α2/α3 isoforms and only α1 activity was observed. Total Na^+^, K^+^-ATPase activity was obtained by adding α1 and α2/α3 isoforms activity [29].

#### 2.4.8. CAT Activity

CAT activity was determined in the liver and the kidneys based on the decomposition of hydrogen peroxide by the catalase present in the sample. This reaction was measured at 240 nm for 120 s in a spectrophotometer and the results were expressed as first-order rate constant k/mg of protein [30].

#### 2.4.9. GST Activity

GST activity was assayed in liver and kidney samples at 340 nm [31]. The reaction occurred when an aliquot of S_1_ was mixed with buffer, GSH, and 1-chloro-2,4-dinitrobenzene (CDNB), which was used as substrate. The activity was spectrophotometrically measured. The results were expressed as nmol CDNB/mg of protein/min.

#### 2.4.10. Western Blot

Western blot analysis was performed according to a previous study by our research group [15]. Primary antibodies were from Santa Cruz Biotechnology (Dallas, TX, USA) anti-phospho-PKCα (1:5000, SC-12356) and anti-total PKCα (1:5000, SC-208). Following overnight incubation, membranes were incubated with the secondary antibodies from Sigma Aldrich (San Luis, MO, USA) (1:10,000, Sigma-A0545). The immunoreactivity was detected with ECL (Thermo Scientific, Waltham, MA, USA) and quantified using the ImageJ software. The phosphorylation ratio was calculated as the relative amount of phosphorylated and non-phosphorylated forms of PKC and normalized by Ponceau.

### 2.5. Statistical Analysis

All data were reported as mean ± S.E.M and normality was assessed using the D’Agostino and Pearson omnibus test. Results were analyzed using GraphPad Prism version 8.0 software and the statistical analysis was carried out by the unpaired Student’s *t* test. A probability of *p* < 0.05 was considered significant. The same software was used for plotting graphs.

## 3. Results and Discussion

The AFB_1_, during its hepatic metabolism, increases the production of reactive species, causing damage to the cell membrane (lipid and protein oxidation) and DNA, altering mitochondrial homeostasis, generating inflammatory responses, and, as consequence, may trigger cell death [32]. Several studies have already demonstrated the damages caused by AFB_1_ exposure, focusing on comprehending the mechanisms underlying its toxic effects [33]. Importantly, no study assessed a less harmful condition for exposure to this toxicant, which is a suitable approach considering the limitations regarding AFB_1_ decontamination [34]. In this context, it is important to highlight that among the various research goals in the field of mycotoxins proposed by the American Phytopathological Society, the one that guides this study is the development of new interventions and prevention strategies against adverse health effects caused by mycotoxins. Thus, we sought to investigate whether the rats submitted to a protocol of intermittent exposure to AFB_1_ would present general toxicity signals, which were assessed by behavioral, biochemical, and molecular approaches.

**Figure 2:**
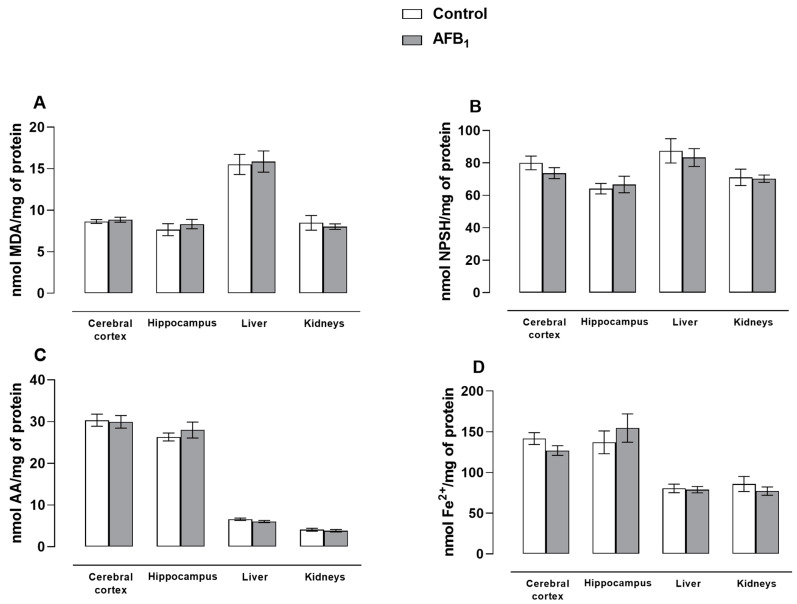
Effect of intermittent administration of AFB_1_ (250 μg/kg b.w., i.g.) or 2% DMSO (10 mL/kg b.w., i.g.) on TBARS levels (**A**), NPSH levels (**B**), ascorbic acid levels (**C**), and FRAP determination (**D**). Data are mean ± S.E.M. for *n* = eight animals in each group. Statistical evaluation was performed by unpaired Student’s *t* test (*p* > 0.05).

The toxicokinetic profile of AFB_1_ estimates a plasma half-life of 36.5 min, renal clearance of 1.25 L/kg/h with approximately 80% of the administered dose excreted within one week [35]. Then, for proper data interpretation, it is important to mention that the selected dose of AFB_1_ used in the animals (250 μg/kg) represents an estimated human dose of 40 μg/kg, which was estimated based on the formula proposed by Reagan-Shaw and collaborators [36]. Disregarding other factors, such as species sensitivity and pharmacokinetics, which could considerably reduce the toxic threshold for humans, the dose of 40 μg/kg means 3.15 mg of AFB_1_ to a human with an average body weight of 70 kg. In this context, the acceptable daily intake of total aflatoxins recommended by the World Health Organization is 20 μg/kg [37]. In this way, such a dose would represent occasional intoxication by high amounts of aflatoxins, since higher doses of AFB_1_ were found in food products (122.35 μg/kg and 2.79 μg/kg in peanuts and cashew nuts, 6.7 mg/kg in peanut products to 36.9 mg/kg in Brazil nuts) [7,38]. Herein, we demonstrated that a protocol of intermittent exposure to AFB_1_ caused no changes in body weight gain (Figure S1), organs weight and relative organ weight compared to the brain weight (Tables S1 and S2), as well as any alteration in the tissue biochemical markers of oxidative stress (cerebral cortex, hippocampus, liver, and kidneys) (Figure 2 and Table 1). The levels of TBARS, NPSH, andAA, FRAP determination, as well as CAT and GST activity did not present statistically significant differences between the control and AFB_1_ groups (*p* > 0.05). Importantly, other studies showed differences in body weight gain 72 h after a single exposition to AFB_1_ (1000 µg/kg, i.g.) [14]. The same AFB_1_ dosage was tested in a repeated administration schedule (seven or fourteen days) to rats in our previous studies and critical hazardous effects to the central nervous system, and hepatic and renal tissues were observed concerning behavioral profile and oxidative tonus [15,18,20,39]. Moreover, changes in oxidative stress were described in several studies that applied higher or lower doses in comparison to the current study [40,41,42]. Thus, our data showed that the schedule of intermittent administration may be an alternative for providing an adequate period for homeostasis reestablishment.

AFB_1_ is known to affect the liver function, causing hepatotoxicity that can progress to liver cancer. As its toxic metabolite is excreted primarily through the urine, changes in renal function are also observed [43]. In this sense, some biochemical parameters were assessed in plasma samples, such as ALT and AST activity, and creatine and albumin content. Moreover, the oxidative status of plasma was investigated as well (FRAP assay and TBARS levels). The results demonstrated that the intermittent exposure to AFB_1_ did not cause any modification in those parameters in comparison to the control group (*p* > 0.05; Table 2). Importantly, these data agree with the other results concerning hepatic and renal tissue, which showed no modification of oxidative tonus. In this context, the repeated administration of AFB_1_ (250 µg/kg/once a day/14 days) increased the hepatic activity of alkaline phosphatase and gamma-glutamyl transferase, while reducing nonenzymatic antioxidant defenses [18], suggesting that the treatment schedule may present low acute toxicity.

Behavioral and biochemical changes in the central nervous system are investigated in models of AFB_1_ exposure given its lipophilic character and its toxic metabolite, which easily cross the blood–brain barrier and are neurotoxic [44,45,46]. The effects of AFB_1_ on the central nervous system are not fully understood, however, it is known that it alters the number and distribution of astrocytes and neurons [47], changes the expression of toll-like receptors, triggering inflammatory responses [48,49,50]. In addition, AFB_1_ exposure decreases brain serotonin, norepinephrine, and dopamine concentration [51], suggesting that this toxicant could impair cerebral functions, such as normal and abnormal behavior, affective disorders, sleep regulation, and cognition, such as learning and memory [33]. Following the intermittent exposure protocol, the animals performed a batch of behavioral tasks to assess anxiety, depressive-like behavior, and memory skills. The results revealed that both experimental groups presented similar behavioral patterns (*p* > 0.05; Table 3), which are reinforced by the cerebral oxidative markers data. As shown in more detail in Table 3, at the end of the protocol chosen, the animals did not present locomotor and exploratory impairment (open field test), short- and long-term memory injury (object recognition test), and induction of anxiety-like behavior (nest construction, marble burying, and splash test). Indeed, while a single AFB_1_ administration (250 µg/kg, i.g.) caused no behavioral changes in rats [18], a repeated treatment schedule (250 µg/kg/once day/7 days) induced locomotor and exploratory impairments and anxiety-like behavior [19]. Overall, our current findings indicate that the interval between AFB_1_ administrations may play a critical role to reestablish organism homeostasis.

**Figure 3:**
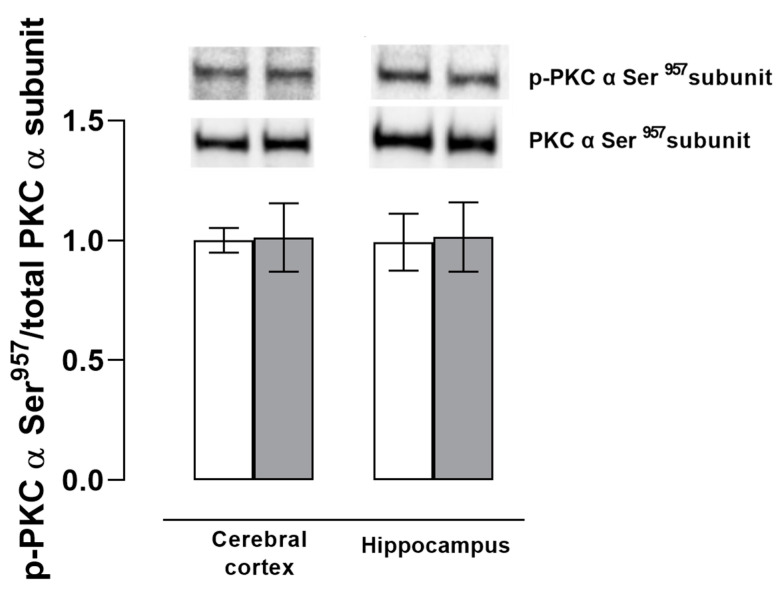
Effect of intermittent administration of AFB_1_ (250 μg/kg b.w., i.g.) or 2% DMSO (10 mL/kg b.w., i.g.) on the phosphorylation ratio of PKC in the cortex and hippocampus. Data are mean ± S.E.M. for *n* = four animals in each group. Statistical evaluation was performed by unpaired Student’s *t* test (*p* > 0.05).

The Na^+^, K^+^-ATPase is a neuronal membrane enzyme that controls the electrochemical gradient, modulates action potential, and the release of neurotransmitters, and is particularly sensitive to oxidative stress [52]. The activity of this enzyme in the cortex was evaluated and no significant differences were observed between treatments (Table S3). Furthermore, it is known that AFB_1_ increases the activation of PKC [18], which is responsible for the processes of cell multiplication and differentiation, morphogenesis, and apoptosis [53]. To investigate the effects of intermittent AFB_1_ on this protein, the immunoreactivity of PKC was estimated by the ratio between the phosphorylated fraction and the total fraction by Western blot. There were no significant differences between the treated and control groups in the cerebral cortex and hippocampus (Figure 3), evidencing that the protocol interval of 96 h is less harmful, with the AFB_1_ chosen dose on the CNS.

Lastly, we must recognize some limitations of this study for proper data comprehension, which are the following: (a) It was applied for investigation of an accessible AFB_1_ dose administrated by intragastric route. Such a route may influence the extent of absorption and metabolism and cause a reduction in the AFB_1_ and its metabolite concentration in tissues. Consequently, it is possible to suggest that the degree and rate of exposure, as well as tissue damage may be also modified; (b) The toxicity of aflatoxins varies according to animal species, age, and sex [54]. Apart from their toxicity to humans, there are species more susceptible to the mycotoxin actions, such as the avian ones [55]. Then, some differences regarding experimental results could be attributed to such parameters; and (c), the absence of a dose-response curve and longer schedule of treatments could limit the mechanistic conclusions regarding the toxic effects of AFB_1_.

## 4. Conclusions

Following an intermittent exposure protocol to AFB_1_, no damage or alteration was observed in the behavior and biochemical markers investigated in rats. These data suggested that in this protocol of exposition to AFB_1,_ it is possible that the animals were able to neutralize the potentially toxic effects resulting from the mycotoxin. Still, regardless of that, AFB_1_ is a highly toxic compound and exposure should be avoided. However, additional studies are required to reinforce our findings and better comprehend the possible outcomes of intermittent exposure to AFB_1_.

## Figures and Tables

**Table 1:** Effect of intermittent administration of AFB_1_ (250 μg/kg b.w., i.g.) or 2% DMSO (10 mL/kg b.w., i.g.) on GST and CAT activity in liver and kidneys.

	DMSO	AFB_1_	*p* Value
GST (nmol CDNB/mg protein/min)			
Liver	61.13 ± 9.43	50.10 ± 8.30	0.39
Kidneys	15.07 ± 2.17	10.13 ± 1.37	0.07
CAT (K/mg protein)			
Liver	1.47 ± 0.15	1.95 ± 0.24	0.12
Kidneys	0.71 ± 0.08	0.59 ± 0.08	0.34

Data are expressed as mean ± S.E.M. for *n* = eight animals in each group. Statistical evaluation was performed by unpaired Student’s *t* test (*p* > 0.05).

**Table 2:** Effect of intermittent administration of AFB_1_ (250 μg/kg b.w., i.g.) or 2% DMSO (10 mL/kg b.w., i.g.) in plasma biochemical parameters.

	DMSO	AFB_1_	*p* Value
ALT (U/L)	48.88 ± 3.44	56.25 ± 4.92	0.23
AST (U/L)	249.30 ± 14.69	284.80 ± 18.78	0.15
CREA (mg/dL)	0.52 ± 0.01	0.52 ± 0.02	0.80
ALB (g/L)	2.95 ± 0.11	2.98 ± 0.04	0.76
TBARS (nmol MDA/mg protein)	3.91 ± 0.21	4.06 ± 0.70	0.83
FRAP (nmol Fe^2+^/mg protein)	11.95 ± 0.36	14.77 ± 1.61	0.11

Data are expressed as mean ± S.E.M. for *n* = eight animals in each group. Statistical evaluation was performed by unpaired Student’s *t* test (*p* > 0.05).

**Table 3:** Effect of intermittent administration of AFB_1_ (250 μg/kg b.w., i.g.) or 2% DMSO (10 mL/kg b.w., i.g.) on the behavioral tests.

	DMSO	AFB_1_	*p* Value
Marble Burying			
Buried marbles (number)	3.75 ± 0.70	4.00 ± 0.82	0.82
Nest Test			
Nest score	4.25 ± 0.36	3.25 ± 0.45	0.10
Splash Test			
Latency to grooming (s)	53.38 ± 6.56	53.00 ± 9.30	0.97
Grooming (s)	115.10 ± 24.11	137.90 ± 18.87	0.46
Object Recognition			
Short-term memory	0.56 ± 0.08	0.59 ± 0.06	0.80
Long-term memory	0.53 ± 0.04	0.48 ± 0.05	0.48
Open Field			
Latency to explore (s)	2.50 ± 0.56	2.25 ± 0.59	0.76
Time spent in center (%)	1.84 ± 0.49	3.03 ± 0.96	0.28
Time spent in periphery (%)	98.16 ± 0.49	96.97 ± 0.96	0.28
Crossings (number)	51.50 ± 4.96	64.50 ± 8.03	0.19
Rearings (number)	24.38 ± 2.79	29.00 ± 5.47	0.46

Data are expressed as mean ± S.E.M. for *n* = eight animals in each group. Statistical evaluation was performed by unpaired Student’s *t* test (*p* > 0.05).

## Data Availability

Not applicable.

## Supplementary Materials

The following supporting information can be downloaded at: https://www.mdpi.com/article/10.3390/brainsci13030386/s1, Figure S1: Effect of intermittent administration of AFB_1_ (250 μg/kg b.w., i.g.) or DMSO 2% (10 mL/kg b.w., i.g.) on body weight gain. Data are reported mean ± S.E.M. for n = 8 animals in each group. Statistical evaluation was performed by unpaired Student’s *t* test (*p* > 0.05); Table S1: Effect of intermittent administration of AFB_1_ (250 μg/kg b.w., i.g.) or DMSO 2% (10 mL/kg b.w., i.g.) on the organ weight of animals; Table S2: Effect of intermittent administration of AFB_1_ (250 μg/kg b.w., i.g.) or DMSO 2% (10 mL/kg b.w., i.g.) on relative organ weight compared to the brain weight; Table S3: Effect of intermittent administration of AFB_1_ (250 μg/kg b.w., i.g.) or DMSO 2% (10 mL/kg b.w., i.g.) on Na^+^, K^+^-ATPase activity in cerebral cortex.
