# Supplementary material for: Intermittent Exposure to Aflatoxin B1 Did Not Affect Neurobehavioral Parameters and Biochemical Markers of Oxidative Stress
Source: Brain Sci. 2023 Feb 23;13(3):386. doi: 10.3390/brainsci13030386 (PMC10046455; doi:10.3390/brainsci13030386)
Supplement: Supplementary file 1 [file brainsci-13-00386-s001.zip › brainsci-2216024-supplementary.pdf]

Supplementary Material

Tables

Table S1. Effect of intermittent administration of AFB_1 (250 µg/kg b.w., i.g.) or DMSO 2% (10 mL/kg b.w., i.g.) on the organ weight of animals.

**Organ**	**Relative weight to body weight**		
	DMSO	*AFB1*	*P value*
Brain	0.97 ± 0.02	0.97 ± 0.01	0.85
Heart	0.44 ± 0.01	0.44 ± 0.01	0.84
Kidneys	1.03 ± 0.02	1.02 ± 0.01	0.85
Liver	5.75 ± 0.19	5.58 ± 0.15	0.50
Lungs	0.72 ± 0.01	0.74 ± 0.02	0.69
Spleen	0.32 ± 0.02	0.35 ± 0.02	0.38
Testicles	0.99 ± 0.03	0.96 ± 0.03	0.57

Data are reported mean ± S.E.M. for n = 8 animals in each group. Statistical evaluation was performed by unpaired Student's t test ($p>0.05$).

Table S2. Effect of intermittent administration of AFB_1 (250 µg/kg b.w., i.g.) or DMSO 2% (10 mL/kg b.w., i.g.) on relative organ weight compared to the brain weight.

**Organ**	**Relative weight to brain weight**		
	DMSO	*AFB1*	*P value*
Heart	45.45 ± 1.71	45.59 ± 1.36	0.94
Kidneys	106.7 ± 2.53	105.5 ± 1.63	0.67
Liver	592.9 ± 13.75	572.8 ± 15.76	0.35
Lungs	75.42 ± 2.65	75.99 ± 2.64	0.88
Spleen	33.42 ± 2.77	36.24 ± 2.79	0.48
Testicles	102.2 ± 3.68	98.39 ± 3.05	0.44

Data are reported mean ± S.E.M. for n = 8 animals in each group. Statistical evaluation was performed by unpaired Student's t test ($p>0.05$).

Table S3. Effect of intermittent administration of AFB_1 (250 µg/kg b.w., i.g.) or DMSO 2% (10 mL/kg b.w., i.g.) on Na^+, K^+-ATPase activity in cerebral cortex.

	DMSO	AFB1	P value	t(df)
**Na^+, K^+-ATPase**				
**(Δ min/mg protein)**				
Isoform α1	50.85 ± 3.55	63.35 ± 8.17	0.18	t(14)=1.40
Isoform α2/α3	21.74 ± 5.08	25.19 ± 2.49	0.55	t(14)=0.60
Total	72.59 ± 8.31	88.54 ± 9.89	0.23	t(14)=1.23

Data are reported mean ± S.E.M. for n = 8 animals in each group. Statistical evaluation was performed by unpaired Student's t test ($p>0.05$).

Figures

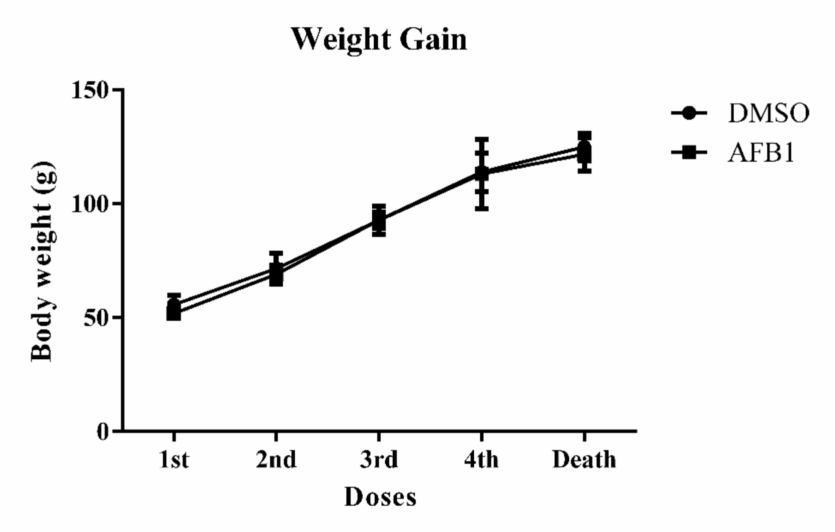

Figure S1. Effect of intermittent administration of AFB_1 (250 µg/kg b.w., i.g.) or DMSO 2% (10 mL/kg b.w., i.g.) on body weight gain. Data are reported mean ± S.E.M. for n = 8 animals in each group. Statistical evaluation was performed by unpaired Student's t test ($p>0.05$).
